# Intrinsic features of the cancer cell as drivers of immune checkpoint blockade response and refractoriness

**DOI:** 10.3389/fimmu.2023.1170321

**Published:** 2023-04-26

**Authors:** Chiara Ursino, Cécile Mouric, Laurent Gros, Nathalie Bonnefoy, Julien Faget

**Affiliations:** Institut de Recherche en Cancérologie de Montpellier (IRCM), Inserm U1194, Univ Montpellier, Institut du Cancer de Montpellier (ICM), Montpellier, France

**Keywords:** immune checkpoint, plasticicity, immunogenicitiy, adjuvanticity, tumor micro environment (TME)

## Abstract

Immune checkpoint blockade represents the latest revolution in cancer treatment by substantially increasing patients’ lifetime and quality of life in multiple neoplastic pathologies. However, this new avenue of cancer management appeared extremely beneficial in a minority of cancer types and the sub-population of patients that would benefit from such therapies remain difficult to predict. In this review of the literature, we have summarized important knowledge linking cancer cell characteristics with the response to immunotherapy. Mostly focused on lung cancer, our objective was to illustrate how cancer cell diversity inside a well-defined pathology might explain sensitivity and refractoriness to immunotherapies. We first discuss how genomic instability, epigenetics and innate immune signaling could explain differences in the response to immune checkpoint blockers. Then, in a second part we detailed important notions suggesting that altered cancer cell metabolism, specific oncogenic signaling, tumor suppressor loss as well as tight control of the cGAS/STING pathway in the cancer cells can be associated with resistance to immune checkpoint blockade. At the end, we discussed recent evidences that could suggest that immune checkpoint blockade as first line therapy might shape the cancer cell clones diversity and give rise to the appearance of novel resistance mechanisms.

## 1 Introduction

The success of immunotherapies, including immune checkpoint blockers (ICB), depend on mutual regulation networks established at the interface between the cancer cells and the non-malignant cell types that constitute the tumor ecosystem. Importantly, the abundance, localization, and functional orientation of each cell component within the tumor microenvironment (TME) vary significantly over time and during treatment. Dissecting and modeling the biological mechanisms governing the TME structuration leaded to the identification of multiple prognostic factors and therapeutic targets, as illustrated by the emergence of ICB as first line therapy in multiple cancer types (Lung KEYNOTE-024, KEYNOTE-189 (1, 2); Melanoma [Chekmate-066) (3); MSIhi-CRC (4)].

PD-1/PD-L1 expression on cancer cells has thought to be important to predict the response to immune-checkpoint inhibitors. However, several studies in non-small cell lung cancer (NSLCLC), confirmed that even if patients showing high expression level of PD-L1 on cancer cells were having the greater survival improvement upon ICB, anti-PD1 immunotherapy was still beneficial across all treated groups, even in patients having moderate to undetectable level of PD-L1 (2, 5, 6).

Tumor immune contexture, representing the pre-existing immune parameters associated with patient survival, appears as a pan-cancer hallmarks linked with the response to ICB. Indeed, high infiltration by CD8 T lymphocytes, B cells, NK cells and the presence of tertiary-lymphoid structures (TLS) are most frequently associated with a better response to therapy. Conversely, massive tumor infiltration by immunosuppressive macrophages, regulatory T lymphocytes (Treg) and polymorphonuclear cells (PMN) such as neutrophils are all linked with treatment failure (7–10). The existence of such common features across multiple cancer types suggest that cancer progression and resistance to treatments might share similar mechanisms to escape the immune control by subverting the host anti-tumor response [for review see: (7, 9)].

Together with immune cell infiltration, alteration of the tumor vasculature is an important factor determining tumor progression and immune contexture. Disorganized tumor vessels hider CD8 T cells trafficking, alter T cell effector functions and might impede dendritic cell maturation. Conversely, T-helper-I (Th1) responses can restore blood vessel normalization and suppress tumor angiogenesis. These observations demonstrated a reciprocal regulation of the tumor vasculature function by the immune stroma while vasculature alteration directly contributes in shaping the tumor immune compartment [for review see: (11)]. Hence, many efforts are currently undertaken to develop novel anti-angiogenic drugs to improve immunotherapies.

Together with endothelial cells, cancer associated fibroblasts (CAFs) account among the most important stromal cells affecting the response to immunotherapy. Notably, single cell analysis of CAF from breast cancer revealed that specific subtypes of CAF would determine the upregulation of (PD-1) and CTLA4 in Tregs. In turn Tregs would mediate Transforming growth-factor β (TGFβ) signaling in CAF contributing to ICB refractoriness (12). In multiple mouse models of solid tumors (pancreas, lung, colorectal and breast), CAFs were shown to form a physical barrier in the tumor mass, by shielding the cancer cell and limiting immune cell ability to infiltrate the tumor mass (13). As an example, Chen et al, recently showed that IL-17 promotes collagen deposition by CAF, enhancing immune exclusion of tumors (14). As illustrated in pancreatic tumors, for which their infiltration is predominant on the TME composition, CAFs from an heterogeneous population of cells with functional diversity ranging from tumor-promoting to anti-tumor activity (15, 16). While both immune cells and endothelial cells are routinely targeted in cancer patients’ care, targeting the fibroblastic tumor compartment did not result in any major translation in clinic yet.

Knowledge describing the relationship between cancer cell clones phenotype and diversity with the TME architecture remains fragmentary. To evaluate the association between the TME and the cancer cell, different kinds of bioinformatics analyses were performed on publicly available databases such as The Cancer Genome Atlas (TCGA). In a recent article, Bagaev et al. (17) studied the tumor immune compartment using a deconvolution data algorithm and co-integrated these signatures with transcriptomic and genomic parameters. This allowed the identification of four distinct TME subtypes conserved over more than 10 000 cancer patients and across 20 different cancers. Finally, this work leaded to the definition of immune favorable TME subtypes displaying the best responses to ICB (17). This research showed that modeling the TME obviously requires a deep characterization of the immune and non-immune tumor stroma, but also of the cancer cell clones phenotype and diversity. A take-home message from these immunogenomics approaches is that specific driver mutations, transcription profiles, microRNA expression, gene copy number variations, metabolic and epigenetic process carried by the cancer cell are involved in the TME structuration (17, 18).

**Figure 1:**
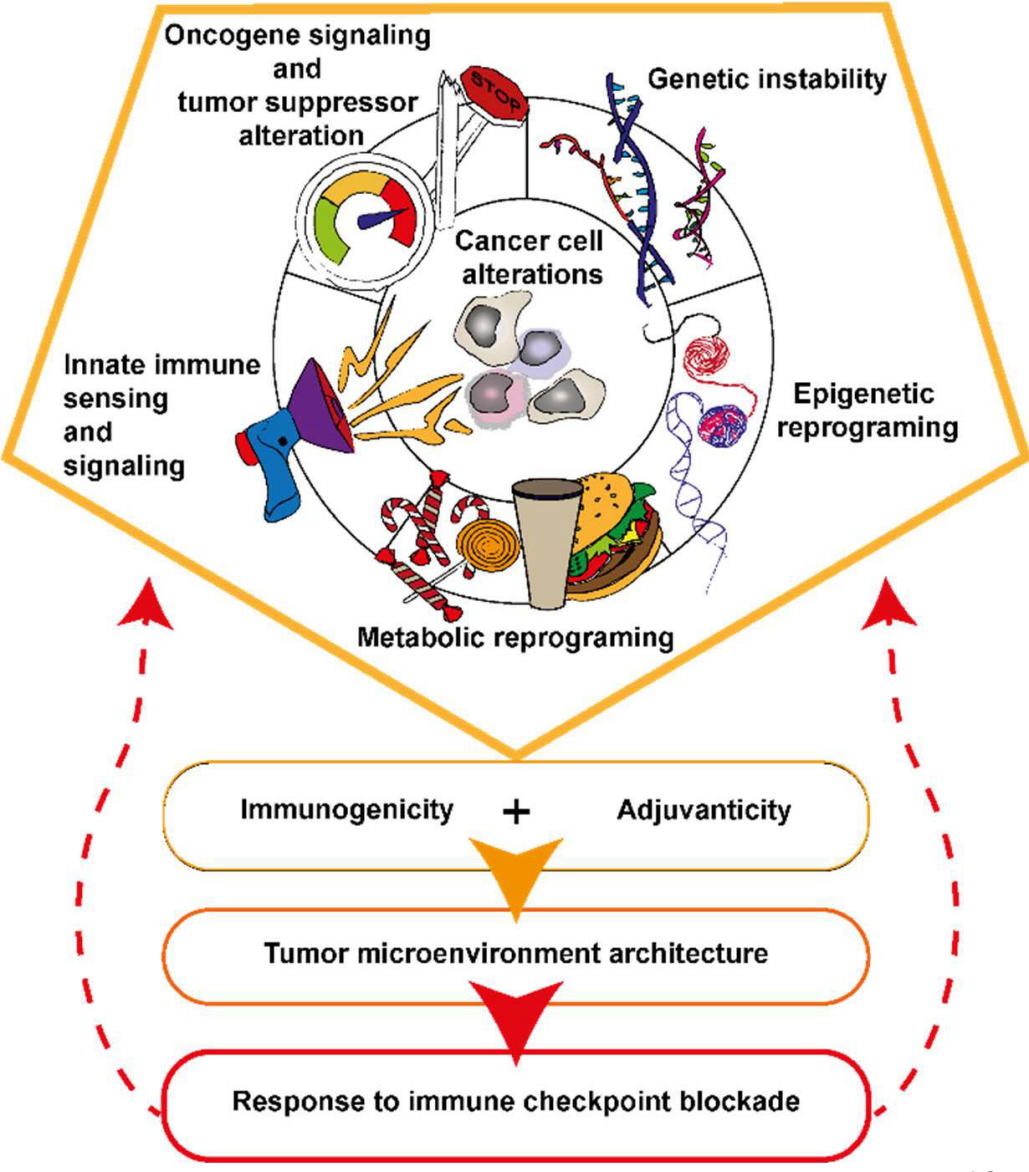
Five types of cancer cell alterations governing tumor immunogenicity, adjuvanticity and the response to ICB. Specificities of oncogenic signaling influence cytokines and chemokine production and ultimately the TME structuration. The nature of the oncogenic and tumor suppressor alteration in the cancer cell associates with genomic instability, epigenetic and metabolic reprograming of the cancer cell. Genetic instability together with epigenetic reprograming are sources of tumor antigens and danger signals accumulation in the cancer cell and TME. Cancer cell undergo metabolic reprograming to sustain their growth, this associates with nutrient deprivation, alarmines and immunosuppressive metabolites accumulation in the TME. Activation or inhibition of innate immune signaling pathways are impacting tumor antigen processing and presentation as well as immune cell recruitment in the TME.

This manuscript will focus on lung cancer and refers to observations showing that specific characteristics of the cancer cells such as the tumor mutation burden (TMB) (19), genomic instability (20), oncogenic stress (21), epigenetics (22), metabolism (23) and intrinsic innate immune signaling (24–26) known to orchestrate the TME architecture and response to ICB (see **Figure 1**). Conversely, the diversity of cancer cell clones forming the tumor is also determined by the host immune system and thus, the TME. Finally, we will discuss whether the expansion of ICB usage in clinic could associate with the emergence of resistance mechanisms.

## 2 Section 1, cancer cell characteristics that might drives immune response

### 2.1 Tumor associated antigens

Non-mutated self-antigens consist of proteins expressed by the cancer cells while normally absent in the healthy tissue and are referred as tumor associated antigens (TAAs). TAAs have been seen as attractive inducers of an antitumor-immune activation. There are different class of TAAs, I) overexpressed TAAs, that are proteins overexpressed in the cancer cells and that, thanks to different mechanisms, can contribute to cancer progression, as MUC-1, HER-2, TERT and Survivin; II) differentiation TAAs, which expression is normally associated to a specific differentiation state of the tissue and that starts to be aberrantly expressed by the cancer cell, like Gp100 in melanoma and the prostatic acid phosphatase (PAP) in prostate cancer; III) cancer testis antigens that are proteins overexpressed in a variety of tumors that should normally be expressed only in immune-privileged germline tissue. Example of this type of antigens are MAGE3, overexpressed in colon, brain, lung and skin cancer, and NY-ESO-1, expressed in a plethora of cancers among which sarcoma, esophageal, ovarian and prostate cancers (27).

Remarkably, PAP-loaded dendritic cell (DC)-based vaccine (Sipuleucel-T) (28) received FDA approval in 2010 (29) for the treatment of castration-resistant metastatic prostate cancer. Ongoing clinical study are evaluating the interest of combining PAP-loaded DC vaccine in combination with ICB (30), yet it is not possible to conclude whether Sipuleucel-T can increase ICB effectiveness in clinic. The efficacy of TTA targeting was also evaluated in a large clinical trial (NCT00480025) on NSCLC patients expressing MAGE-A3 treated with MAGE-A3 vaccine (AS15 proprietary adjuvant). which failed showing any benefit (31). Similar results have been shown in a study on different solid cancers using NY-ESO-1 based vaccine. Indeed, no activated CD8 T cells nor increased levels of antibodies against NY-ESO-1 were detected in patients’ blood and, coherently, responses were similar between treated and control arms of the study (32). An interesting study conducted by Cebon et al., demonstrated that the absence of clear therapeutic response in patients treated with NY-ESO-1 based vaccine could be associated to the reduction of HLA-NY-ESO-1 complexes in the cancer cells as a mechanism of resistance (33). Multiple DC-vaccines were or are being evaluated in melanoma based on gp100 and MART1 TAAs. The combination of a DC vaccine with ICB has been shown to be effective in treatment of melanoma patients. Even after recurrence in patients who received adjuvant DC vaccination, treatment with first- or second-line PD-1 inhibitor monotherapy resulted in a response rate of 52% suggesting that adjuvant DC vaccination might increase ICB effectiveness at least in recurrent disease (34). However, the clinical benefit from TAA vaccination remains limited. This might be linked to the fact that TAAs are self-antigen against which the generation of a strong immune response is avoided thanks to central and peripheral immune tolerance. Therefore, the manipulation of the immune compartment concomitantly to TAAs delivery may be needed to assure clinical efficacy of TAA based vaccine strategies. The efficacy of the PAP-loaded-DC-based vaccines is an example, patient immunization with dendritic cells modified to express PAP fused to granulocyte-macrophage colony stimulating factor (GM-CSF), to promote DC survival and proliferation, is able to overcome immune tolerance and generate a proper immune response against prostatic cancer (28).

### 2.2 Genomic instability and tumor specific neoantigens

Genomic instability, defined by an increased tendency for DNA mutations and other genetic changes during cell division, translates into a higher tumor mutational burden (TMB, number of somatic mutations per megabase) (35), is thought to be linked with cancer cell immunogenicity and anti-tumor immune response. Indeed, it is considered as important for the accumulation of tumor-specific neoantigens (TSA) in the cancer cells, which are produced by mutation in exonic sequences and can constitute the substrate of the adaptive anti-tumor immunity as they are exempted of central tolerance (36). Cancer cell TSA load appears as a critical factor linked with ICB clinical benefit (37, 38), as most of the cancer types for which ICB were approved as first line treatment show remarkably high TMB (ranging from 5 to 20 mutations/megabase) (36). However, the association between TMB and response to ICB among patients suffering from the same cancer type is not always so evident. In colorectal cancer (CRC), microsatellite-instability-high-CRC (MSI^hi^-CRC) show greater sensitivity to ICB (approved as first line treatment) when compared to microsatellite stable-CRC (MSS-CRC) upon ICB treatment. First line ICB was approved for mismatch-repair-deficient (dMMR) and MSI^hi^-CRC [for review see (39)]. Interestingly, immunopeptidomic analyses performed on MSI^hi^ and MSS-CRC did not identify more putative TSA in the MSI^hi^ group (40) when compared to MSS-CRC samples. At the molecular level, other major differences exist between CRC subtypes and could explain differences in ICB sensitivity through mechanisms independent of TSA generation. Nevertheless, in the case of non-small-cell lung cancer (NSCLC), a better response to ICB was reported among patients harboring mutations in a series of genes implicated in DNA replication and repair as POLD1, POLE, MSH2, DNA-PK and RAD17It (41). A more recent study performed on 12 different types of cancer established that around the 70% and 21% of patients were showing respectively partial and complete responses since they were displaying defect in the DNA damage repair (DDR) machinery and the expansion of TSA specific CD8 T cell-clones was observed in ICB responder patient (42). Wang and colleagues demonstrated the crucial importance of DNA damage repair defects, more specifically co-mutation on homologous recombination repair and mismatch repair (HRR-MMR) and homologous recombination and base excision repair (HRR-BER), and their association with higher TMB, neoantigen load and immune-regulatory gene expression. In this study, defect in HRR-MMR and HRR-BER represents a predictive biomarker for patients’ response to ICBs. Interestingly, this work underline that the key element to have enhanced efficacy of immunotherapy response is the concomitant mutation of two important mismatch repair pathways, since mutations caused by a defect in a single DDR pathway determine a low TMB and TSA load, not high enough, to be predictive of the outcome of therapy (43). Finally, together with DNA repair defect, mutations in tumor suppressor genes important for genome integrity, as Tp53, were associated with higher TMB and response to ICB in NSCLC and head and neck squamous cell carcinoma (HNSCC). (44–46). However, a direct association between TMB, the TSA load and the T cell repertoire, as predictive factor of the response to ICB among patients suffering from the same pathology remains to be better understood and characterized. While homologous recombination deficiency was associated with a better survival in high grade serous ovarian cancer, this parameter did not strongly correlate with T-cell receptor clonality even if the latter showed strong positive link with patients’ survival (47). Furthermore, the TMB is not always associated with better outcome of ICB. For example, in a study on renal cell carcinoma (RCC) patients treated with PD-L1 or combinational therapy of PD-L1 + anti-VEGF-A, the TMB and the tumor neoantigen burden (TNB) did not correlate with the response to therapy. The reason behind this might come from the fact that not all mutations can give rise TSA capable of supporting the anti-tumor adaptive immunity. The evaluation of nonsynonymous mutation burden is a better predictor of immunotherapy response than total exonic mutation burden (41, 44). Neoantigens derived from gene fusion can also provide a pool of immunogenic TSA in TMB low cancer. This phenomenon was nicely exemplified in HNSCC, with the description of a patient showing a complete response to ICB. This patient displayed a tumor with very low CD8 T cells infiltration, low TMB and low PDL1 expression before ICB, but presented a DEK-AFF2 fusion resulting in an HLA-C restricted TSA for which specific T cells were detected in the patient’s blood (48). These findings might be of importance for many types of cancer characterized by gene fusion, thus important efforts have to be done in improving tools for detecting gene fusion and predicting immunogenic TSA load (49, 50).

Recently, a second family of tumor specific antigen was identified from immunopeptidomic. These are the non-mutated aberrantly expressed (aeTSA), which, emanate from the transcription and translation of non-coding genomic regions and represent the majority of the TSA pool (40, 51). Furthermore, about 3,000 transcriptionally active endogenous retroviruses (ERVs) were identified in the TCGA dataset across multiple cancer types, and ERVs signature has been used to predict immunotherapy response (52) since their expression can associate with expression of aeTSA (52) and therefore with cancer cell innate immune signature. Interestingly, not only the ERVs, but also other kind of transposable elements (TEs) such as long interspersed nuclear elements (LINEs), short interspersed nuclear elements (SINEs) can become a source of aeTSA (53). The current consensus suggests that expression of these aeTSA is mainly due to epigenetic dysregulation in the cancer cell (52–54).

Future development of personalized cancer treatments will most probably benefit from the emergence of RNA based vaccines allowing the design of a specific TAA/TSA cocktail for each patient. The development of such strategies will require improving our ability in identifying the best tumor antigens that should also include aeTSA. However, tumor antigen based therapeutic strategies requires that cancer cells indeed express and present these antigen on their major histocompatibility complex-1. Cancer cells can control their immunogenicity through divers alterations of MHC-I expression linked with IFN sensing pathways as well as antigen processing and peptides loading on MHC-I molecules. These mechanisms were described in studies of mechanisms linked with ICB insensitivity. In this review, they will be shortly evoked in the discussion as they were also observed in the context of acquired resistance to ICB treatment [for review see, (55)].

### 2.3 Cancer cell epigenetics and antiviral-like innate immune signaling

Genetic mutations are not the only genomic alterations that ultimately drive antitumor immune-surveillance. Structural alterations, like epigenetic modifications, represent a cancer cell-intrinsic factor that modify the TME as well. Indeed, epigenetic modifiers were found to be important in cancer responsiveness to ICB. Beside from driving the expression of TSA and TAA, the deregulation of the epigenome can also lead to the accumulation of nucleic acids into the cytoplasm ultimately sensed by innate immune signaling pathways into the cancer cells. An example of this phenomenon is the enhanced bidirectional transcription of ERVs caused by the inhibition of different histones demethylases. ERVs would then fold in double stranded RNA (dsRNA) that can be sensed in the cytoplasm by toll-like receptor 3 (TLR3), melanoma differentiation-associated gene 5 (MDA5) and IRF7 initiating an antiviral-like innate immune cascade ultimately driving interferon (IFN) signaling activation and tumor growth control. This phenomenon has been shown in human in *in vitro* models of breast, kidney, skin, lung (56), ovarian (57) and colorectal cancers (58). The opportunity to trigger cancer cells innate immune signaling was pharmaceutically exploited to remodel the immune compartment into the tumor mass. Indeed, therapeutic strategies that implies the coupling of methyltransferase inhibitors and immunotherapy result in increased anti-PD1 (56) and anti-CTLA-4 (57) responses in mouse models of melanoma. Together with ERVs, small non-coding RNA enriched in ds-structures, like U1 and U2 and long non-coding RNA (lncRNA) can lead to the activation of the of retinoic acid-inducible gene I (RIG-I) (59). This was exemplified through the characterization of long non-coding RNA antisens (lnc-RNA-AS) which can activate the IFN response leading to an anti-proliferative activity at least in part via interferon regulatory factor 1 (IRF1) in esophageal squamous cell carcinoma (60). Similarly, RNA binging proteins (RBPs), that have important functions in mRNA splicing and stabilization, seem to be interesting pharmaceutical targets to enhance innate immune response trough similar mechanisms. For instance, in two *in vitro* models of breast cancer the repression of heterogeneous nuclear ribonucleoprotein C (HNRNPC), a RBP, generated pre-mRNA introns that gave rise to dsRNA highly enriched in Alu elements belonging to HNRNPC binding sequences. This was shown to trigger RIG-I signaling therefore determining IFN response and tumor growth arrest (61). Thus, epigenetic alteration together with endogenous innate immune signaling in the cancer cell represents a very attractive therapeutic opportunity [for review see (54)].

## 3 Section 2: cancer cell characteristics linked with ICB refractoriness

### 3.1 Metabolic pathways linked with ICB resistance

**Figure 2:**
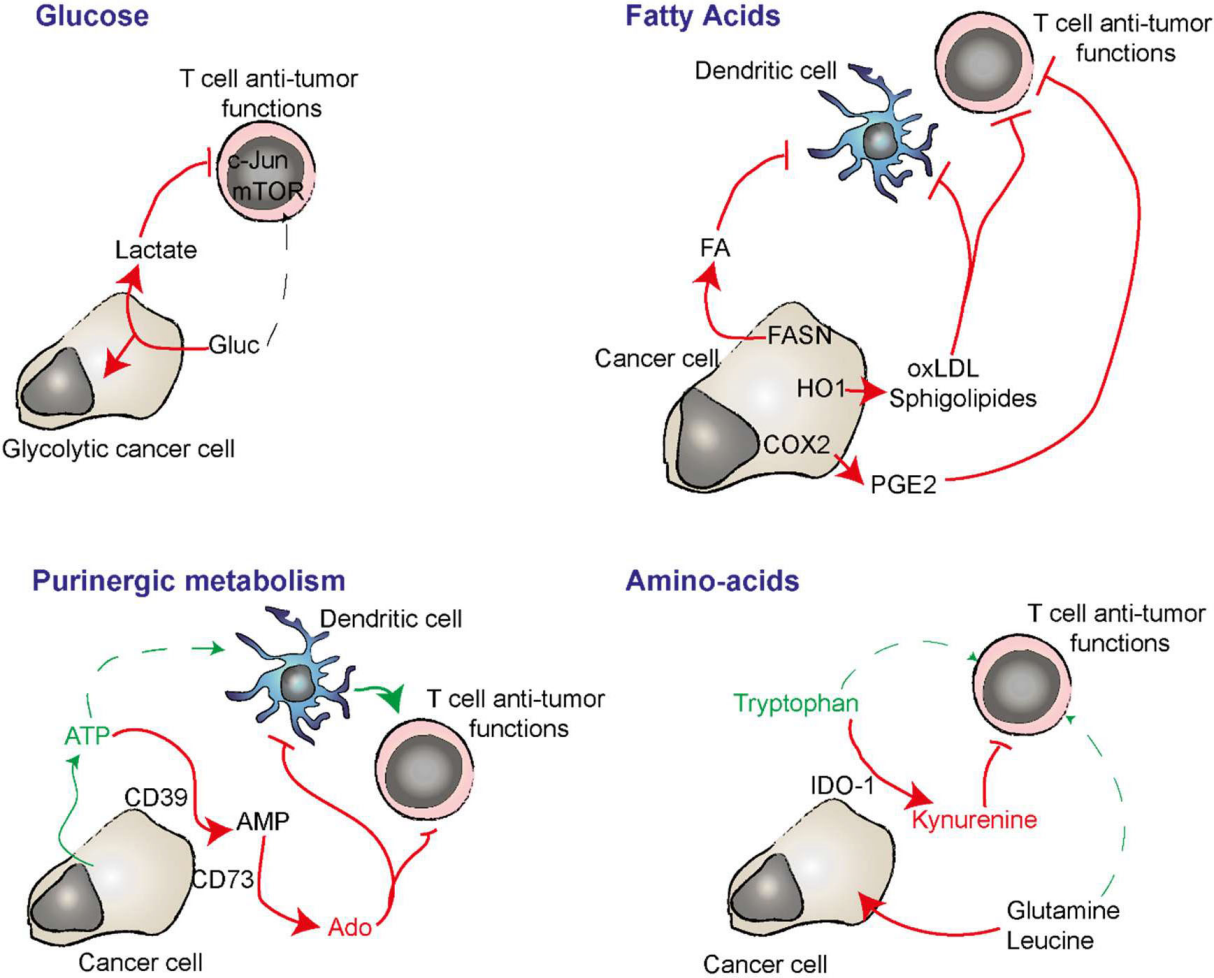
Nutriment deprivation, immunosuppressive metabolites and regulation of purinergic signaling are important players in cancer cell immunosuppression and adjuvanticity. Cancer cells have a strong avidity for glucose which lead to it deprivation in the TME while T cells requires it fo optimal anti-tumor function. In absence of oxygen, glycolytic cancer cells produce lactate from glucose. Lactate can have an immunosuppressive effect on T cells. Cancer cells can synthetize important amount of immunosuppressive lipids by expressing FANS. HO1 can oxidize LDL that have an immunosuppressive impact on DC and T cells. COX2 converts arachidonic acid into immunosuppressive PGE2. CD39 and CD73 expressed by the cancer cell degrade extracellular ATP into adenosine (Ado). ATP is a danger signal able to induce inflammatory while adenosine inhibits T cell activation and favors DC and macrophage immunosuppressive functions. IDO-1 converts pro-inflammatory extracellular Tryptophan in to immunosuppressive kynurenine. Cancer cell avidity for glutamine and leucine deprive the TME in these amino acids that play important roles in anti-tumor T cell polarization and function.

Cancer cells affect the immune microenvironment through the metabolites that they release into the TME that can have a detrimental effect to the bystander cells, or through competition for crucial nutriments. Indeed, immune cells antitumor functions, which includes local T cell proliferation, motility and the production and release of cytokines and chemokines, require high amounts of energy. For example, upon activation, T cells increase their oxygen consumption since they boost up aerobic glycolysis and glutaminolysis (62). Thus, glucose deprivation results in a massive impairment of T cell functions. Consistently, in a mouse model of sarcoma, the tumor glucose consumption dampened T cell mammalian target of rapamycin complex 1 (mTORC1) activity, their capacity to do glycolysis and to produce IFNγ, lowering adaptive anti-tumor activity in spite of the high antigenicity of the cancer cells (63, 64) (see **Figure 2**).

#### 3.1.1 Anaerobic and aerobic metabolism of the cancer cell and immunity

In anaerobic conditions, the enhanced glycolysis associates with the accumulation of pyruvate which is then converted into lactate by lactate dehydrogenase A (LDH-A). This enzyme is particularly active in KRAS and EGFR mutant tumors, where its inhibition determines reduced glycolytic flux *in vivo*, *ex vivo* and *in vitro* and decreased lactic fermentation. This type of metabolism seems to be essential for lung cancer-initiating cells and disease progression, since inhibition of LDH-A results in tumorigenesis impairment and regression of already established tumors (65). High glucose consumption and lactate production and excretion is common to different types of cancers resulting in the acidification of the TME. An environment particularly enriched in lactic acid is detrimental for the immune microenvironment. In particular T cells exposed to lactic acidosis have impaired phosphorylation of c-Jun N-terminal kinases (JNK), C-JUN and p38 which drives the blockage of cytokines production, proliferation (66) and the general impairment of the effector CD8 T cells functions (67). Coherently, high lactic acid levels in the blood of metastatic lung cancer patients are predictor of poor survival (68).

In presence of oxygen, pyruvate can also be converted to Acetyl Co A that fuel the TCA cycle for the production of high quantity of energy. In KRAS and KRAS and Liver kinase B1 (LKB1) (KL) mutant tumors the oxidative phosphorylation seems to be another metabolic process importantly enhanced (69, 70). This process requires high amount of oxygen for the tumors cells, and limiting oxygen availability within the TME affects immune cell polarization. Indeed, oxygen is critical for T cell differentiation and activity upon immunotherapy. In melanoma, *in vitro* and *ex vivo* experiments from patients’ samples showed that tumor oxidative metabolism determines elevated tumor oxygen uptake from the TME that was associated to T cells exhaustion and therefore decreased immune activity and anti-PD1 response (64). While it might sounds difficult to target cancer cell metabolic profile without strongly affecting heathy and stromal cells function, retrospective studies highlighted the fact that Metformin, a component used to treat diabetes seems to increase ICB efficacy in patients from multiple cancer types. The mechanisms through which it might work remains to be better understood yet but the impact on cancer cell metabolism seems obvious, thus multiple clinical trials are ongoing [for review see (71)].

#### 3.1.2 Fatty acids metabolism of the cancer cell and immunity

Aside from the TCA cycle, glucose metabolites derived from enhanced glycolytic flux are often redirected toward citrate synthesis, an important intermediate for lipid biosynthesis. This happens in KRAS mutant cancer, where hexokinase 2 (HK2) overexpression has been shown to be a key player to divert glucose in ribonucleotide and fatty acids synthesis (70). This was also observed in LKB1 mutant lung cancer in a process supported by mTORC1-dependent Hypoxia-inducible factor 1-alpha (HIF1a) expression (69, 72). These anabolic pathways are important for production of biomolecules that assure cancer cell proliferation. Indeed, fatty acids synthesis is needed for the growth of NSCLC. Inhibition of ACC (Acetyl CoA Carboxylase), one rate limiting step of *de novo* fatty acid synthesis, reduces tumor growth in KP and KL mouse models (73). Nevertheless, the fatty acids synthase (FASN) is overexpressed in NSCLC and its inhibition leads to a diminution of lactate and ATP production, as well as cancer proliferation, invasion and migration (74). Interestingly, fatty acids synthesis, in addition to be crucial for cancer progression, is also influencing the TME. Indeed, in ovarian cancer FASN expression has been associated with an immunosuppressive immune microenvironment. FASN activity in cancer cells is responsible for the accumulation of fatty acids in the TME, which are detrimental for DC ability to present antigens. Inhibiting FASN was partially restoring the immunostimulatory ability of the DC in a mouse model of ovary carcinoma (75). In NSCLC, bioinformatics analyses of the TCGA and of a cohort of 240 patients treated with ICB showed that mutations in genes involved in fatty acids metabolism associate with a greater response to therapy. Interestingly, patients displaying highest mutation rates in fatty acids metabolism genes are also those showing higher PDL1 expression and TMB in cancer cells (76). Cancer cell metabolism alterations associate with perturbations of cholesterol synthesis (77), oxidized low-density-lipoproteins (ox-LDL) through cancer cell expression of the heme oxygenase-1 (HO-1) (78), and sphingolipids diversity and accumulation (79). These perturbations of lipid biosynthesis and metabolism occurring in the cancer cells were shown to affect T cells and antigen presenting cells anti-tumor functions in multiple cancer types, and might contribute to ICB refractoriness. Finally, another link between cancer cell fatty acid metabolism and response to ICB resides on the expression of the cyclo-oxygenase-2 (COX-2) that leads to the accumulation of prostagandine-E2 (PGE2) produced from polyunsaturated fatty acids (arachidonic acid). Indeed, COX2 expression by the cancer cells was suggested to be an important immune escape mechanism. While PGE-2 induces blood vessel dilatation and inflammation, COX2 expression by the cancer cells was shown to inhibit T cell accumulation in the tumor mass, to favor immunosuppressive myeloid cell polarization, to impair DC and NK cell functions and to drive the expression of the indoleamine 2,3-dioxygenase-1 (IDO-1) [for review see (80)]. Thus, COX2 specific inhibitors such as celecoxib were evaluated in clinic in combination with targeted therapy, chemotherapy and ICB. Unfortunately, this failed demonstrating clear benefit for the patients yet, but numerous clinical trials are still ongoing (NCT03026140, NCT03926338, NCT04188119, NCT04348747). However, non-specific COX2 inhibitor such as Aspirin and non-steroidal anti-inflammatory drugs (NSAID) are frequently administrated to cancer patients receiving ICB and retrospective analyses provided contradictory results making impossible to conclude if COX2 inhibition would be or not beneficial in combination with ICB at least for lung cancer (81, 82).

#### 3.1.3 Amino-acids metabolism in the cancer cell and immunity

Amino-acids degradation within the TME can be unfavorable for the immune compartment. Almost all cell types in tumors including the cancer cells can express IDO-1 that degrades tryptophan into kynurenine. IDO-1 expression by the cancer cell was associated with immunosuppressive function through inhibition of T-cell receptor expression and induction of Treg [for review see (83)] andhigh IDO-1 activity was linked with ICB refractoriness in NSCLC (84), therefore making it a very attractive target and multiple inhibitors have been developed and are under evaluation in multiple cancer types. However, the phase III clinical study of epacadostat a selective IDO-1 inhibitor in combination with pembrolizumab in melanoma patients (ECHO-301/KN-252) provided negative results (85). Indoximob, another IDO-1 inhibitor was recently evaluated in combination with ICB in a phase II clinical trial on melanoma patients. However, while the percentage of patients showing disease control was encouraging, this was a single arm study, making impossible to conclude whether the combination therapy performed better that ICB alone (86). Aside from tryptophan degradation, LKB1 mutant tumors have a pro-growth metabolism characterized by high glutamine uptake and usage (72). Similarly, in KRAS mutant NSCLC, oncogene activation drives the uptake of amino acids, such as glutamine and leucine in a phosphoinositide 3-kinase (PI3K)/Nuclear factor erythroid 2-related factor 2 (NRF2)/Activating transcription factor 4 (ATF4) dependent mechanism that leads to a pro-oncogenic metabolism reprogramming (87). The scarce availability of glutamine in the TME determine CD4 T cell polarization toward Foxp3^+^ Tregs because of decrease in mTORC1 signaling, regardless of the presence in the TME of cytokines that would normally lead to Th1 differentiation (88). Oh and colleagues showed in a mouse model of triple negative breast cancer resistant to immunotherapy, that inhibition of glutamine metabolism coupled with a-PD1, led to the sensitization to immunotherapy thanks to the consequent immune compartment reprogramming, with the increased activation of macrophages and antigen presentation as well as the death of myeloid derived suppressor cells (MDSCs). However in this study total glutamine metabolism was blocked in both cancer and stromal cells, thus it is not possible to descriminate to what extend glutamine uptake and usage specifically by cancer cells might interfere with ICB (89). These evidences open the doors to new attractive pharmaceutical possibilities to bypass immunotherapy resistance.

#### 3.1.4 Cancer cell purinergic signaling and immunity

Another metabolite that importantly impacts tumor growth and drives immune escape is adenosine. Adenosine is produced through extracellular ATP degradation mainly via two ectonucleotidases, CD39 and CD73. While ATP release in the TME consequent to cellular stressing events as hypoxia and necrosis constitute an important alarmin favoring antigen presenting cell recruitment and T cell activation, adenosine signaling can fuel tumor growth and metastasis formation while impairing T-cell receptor (TCR) signaling and favoring immunosuppressive myeloid cell functions (23). In lung cancer, epithelial growth factor receptor (EGFR) mutant tumor cells have been shown to express high levels of CD73, which give rise to high concentrations of immunosuppressive adenosine in the TME (90, 91). Recently, a subclass of NSCLC stem-like cells, expressing CD133 and CXCR4, were shown to express CD38, PC-1 and CD73, enzymes that, leads to the production of high level of adenosine through the non-canonical adenosine pathway (92). Since NSCLC patients refractory to aPD1 immunotherapy show high expression of adenosine receptor (93), clinical interest is aroused regarding the inhibition of adenosine signaling. In *in-vivo* model of melanoma and NSCLC the inhibition of adenosine receptor with specific antagonists suppresses tumor cell growth and induced the expression of PD-L1. Coherently, the coupling of aPD-L1 and adenosine receptor antagonist gave promising synergistic effects (93). An interesting phase I clinical trial on metastatic or advanced NSCLC patients is actually ongoing to evaluate the safety of on PBF-1129, a selective Adenosine A2b receptor antagonist (NCT03274479). Similarly, early clinical evaluation of CD73 and CD39 inhibitors are ongoing (NCT04148937, NCT05075564).

Metabolism reprogramming occurring in cancer cells represent an important mechanism of TME reshaping, immune escape and resistance to immunotherapy. Thus, it might generate new therapeutic avenues to predict and enlarge immunotherapy sensitivity. Research for extending current knowledge on how cancer cell metabolism could be manipulated to boost immune activation is an expanding field, although yet it failed delivering significant improvement of patient’s care. Because they are intrinsically linked together, characterization of oncogenic signaling and targeting of cancer cell metabolic subtypes needs to be integrated toward enhanced anti-tumor immunity.

### 3.2 Oncogenic signaling and ICB refractoriness

Studies in lung cancer show remarkable evidences that the type of oncogenic signaling and tumor suppressor alteration are tightly associated with the response to ICB. Similarly, specific mutations linked with cancer cell oncogenic signaling were shown to associate with survival of melanoma patients upon ICB (94). In this section, we will see that oncogene and tumor suppressor alterations can drive different outcomes on the tumor ecosystem directly through specific signaling events or by interfering with cancer cell genomic stability and metabolism.

#### 3.2.1 KRAS

KRAS mutated lung cancer have been linked with incremented glycolytic flux (95) and this is also the case also for EGFR mutant tumors, in which the activation of PIP3K/AKT/mTORC1 supports glycolysis and consequently fosters cell proliferation (96). Inhibition of PI3K/mTOR in these tumors decreased the membrane localization of the glucose transporter 1 (GLUT1) (96) which expression in lung adenocarcinoma has been associated with adverse clinical outcome, lymphovascular invasion and advanced TNM stage (97). However, KRAS mutant tumors often display co-occurring mutations, among which TP53 is the most common. As previously discussed, TP53 mutations are linked with a higher TMB of the cancer cells. Thus, KRAS-TP53 co-mutated (KP) cancers are those that demonstrated the best response to immunotherapy (98) having high TMB, high expression of PD-L1 that seems to depend on extracellular signal-regulated kinases (ERK) signaling (99) and high T cell infiltration (100, 101). Importantly, specific KRAS mutation might be associated with different expression levels of PD-L1 in NSCLC cells. Indeed, PD-L1 is expressed at higher levels in KRAS^G12D,G12V,G13C^ mutated cancers and at lower levels within KRAS^G12A^ and ^G12C^ mutated cancers (102), suggesting that different KRAS mutations confers to the mutated protein higher or lower ability to influence downstream signalling pathways that then shapes the TME.

#### 3.2.2 EGFR

Activating mutations on EGFR, are often present in NSCLC cells. Treatment with tyrosine kinase inhibitors (TKI) are the standard of care in this case, but almost systematically, resistant mechanisms will appear. The use of immunotherapy on EGFR mutant patients was recently evaluated but it gave poor outcome, suggesting that EGFR oncogenic signaling could represent an intrinsic resistance mechanism to immunotherapies. Indeed, analysis of NSCLC patients harboring or not EGFR mutations revealed that EGFR mutant tumors are not responding to immunotherapy irrespectively of their PD-L1 expression levels (103, 104). One of the reasons for such resistance could be the fact that these tumors show low TMB and expression of immune checkpoints (90, 105). Furthermore, the analysis of the immune microenvironment of EGFR mutant tumors highlighted a TME enriched in immunosuppressive cell types (106) and displaying low T cell clonality and CD8 T cell density. Such immunosuppressive TME could be explained by the tight regulation of important chemokines for the recruitment and the activation of immune cells in EGFR mutant cancer cells. Indeed, compared to the WT, EGFR mutant cancer cells downregulate the expression of CXCL10, chemoattractant for CD8 T cells, and CCL21, important for the accumulation of NK cells and naïve T cells, and whole tumor extracts revealed a trend toward a lower expression levels of IFNγ (90, 107). Moreover, EGFR mutant cancer cells upregulates TGFβ and CCL22 that are important for Tregs polarization and accumulation, and CXCL8, a neutrophil chemoattractant (90, 107). Finally, aberrant EGFR pathway activation would cause a suppression of IRF1 signaling and therefore, even when stimulated with IFN, EGFR mutant cells upregulate PDL1 at a lower extent compared to their WT counterpart (107). These data describing an inert immune compartment in EGFR mutant cancers could explain the refractoriness of these tumors to immunotherapy.

#### 3.2.3 Phosphatase and tensin homolog (PTEN)

Analyses of immunotherapy resistant gliomas, leyomiosarcomas and melanomas revealed that resistant phenotypes are in some case associated to the loss of PTEN (108–110). Indeed, PTEN loss in cancer cells determines the constitutive activation of PI3K-AKT pathway, which in turn causes the upregulation of several cytokines that shape the surrounding TME (109). Specifically, in patients affected by melanoma and metastatic leyomiosarcoma, PTEN loss was demonstrated to contribute to immunosuppressive cells infiltration in tumors, while avoiding lymphocytes accumulation (108, 109). The inhibition of PI3K, when combined with anti-PD-1 in syngeneic model of PTEN deficient melanoma resulted in an increased efficacy of immunotherapy, associated with an increase of CD8 T cells infiltration and efficient tumor regression (111). In lung cancer, PTEN loss has been associated to lower patients’ overall survival (112, 113). In *in vitro* models of lung adenocarcinoma, PTEN loss results in the activation of the ROS/SHP2 pathway, ultimately leading to unresponsiveness of these cells to IFNγ signaling (114), suggesting that in lung cancer PTEN loss and PI3K activation are also associated with lower responses to immunostimulating therapies.

#### 3.2.4 Anaplastic lymphoma kinase

Alteration of the of Proto-oncogene tyrosine-protein kinase (ROS), Rearranged during Transfection (RET) and ALK genes have been associated with ICB refractoriness (101). Specifically in ALK rearranged tumors, the resistance to immunotherapy seems to be link to the low PD-L1 expression and the lack of CD8 T cells (115, 116) and activated memory CD4 (116). Interestingly, ALK rearrangement in lung cancer, via augmented PI3K/AKT pathway activation, have been associated to an important expression and activation of HK2), a key enzyme of the glycolysis, that contributes to high glucose usage via aerobic glycolysis (117). However, the mechanism behind this deleterious shaping of TME remains poorly understood.

#### 3.2.5 WNT/β-CATENIN

In several type of cancers, including lung cancer, the activation of WNT/β-catenin signaling in cancer cells has been associated with T cell deprivation in the tumor mass, low PD-L1 expression and resistance to immunotherapy (118–120). In both colorectal cancer and melanoma, the activation of β-catenin/AMP-dependent transcription factor (ATF3) signaling inhibits CCL4 secretion by the cancer cells, a crucial chemokine for the recruitment of DCs in the tumor mass, and therefore the correct priming of a potent antitumor immune response (121, 122). The absence of DCs was linked with naïve T cells accumulation in the tumor mass, that are not proliferating nor having cytotoxic function even if cancer cells showed high tumor antigen expression (122). Interestingly, β-catenin has also been shown to be activated in metastatic lesions and participate to acquired resistance mechanism to immunotherapy (123, 124). This was illustrated by the case of a melanoma patient, in whom the primary tumor showed a good response to immunotherapy but who developed a metastatic lesion with acquired resistance to immunotherapy. Despite the presence of circulating T cell against TSAs expressed by the cancer cells of the metastatic lesion, these T cells failed accumulating in the metastatic site, most probably because of acquired constitutive activation of β-catenin signaling in metastatic cancer cell clones (124). These notions suggest that during the cancer progression the tumor cells acquire the ability to activate b-catenin signaling as a mechanism of escape from immune surveillance. The cancer cells would therefore go through a change in their phenotype to survive trough out therapy, a process that raise questions on how cancer cell plasticity could be used as a mechanism of resistance to therapy.

#### 3.2.6 STK11/LKB1

An integrative analysis of genomic, transcriptomic and proteomic data on KRAS mutated lung adenocarcinoma patients shown that the most frequent co-occurring genomic alterations, together with KRAS mutation, are the loss of STK11/LKB1 or TP53 functions. Importantly, the high sensitivity of KRAS mutated cancers to ICB is lost if cancer cells harbour co-mutation in the tumor suppressor STK11/LKB1 instead of TP53 (101, 125–127). The alteration in STK11/LKB1 or TP53 were clonal and non-overlapping among cancer initiated by KRAS amplification, suggesting that they are mutually exclusive (125). This notion arises the possibility that KRAS-LKB1 (KL) mutant cancer are refractory to immunotherapy rather because they usually preserve unaltered expression of TP53 than because of intrinsic LKB1-dependant mechanisms. Indeed, LKB1 mutant or deprived tumors have lower TMB, low tumor-infiltrating CD8 T cells and low PD-L1 expression when compared to KP (125). However, low expression of PD-L1 might not be the major criteria for the impaired response to immunotherapy, since a retrospective study on NSCLC patients reveal that LKB1 mutant tumors that have high PDL1 expression are also refractory to ICB (125). Koyama and colleagues that studied the TME in a mouse model of NSCLC mutated for KRAS and LKB1 have proposed a mechanistic explanation for the resistance to immunotherapy in these tumors. They found that LKB1 mutant cancer cells, in addition to having decreased PDL1 levels, showed an upregulation of pro-inflammatory cytokines as chemokine (C-X-C motif) ligand 7 (CXCL7), granulocyte colony-stimulating factor (G-CSF) and interleukin 6 (IL-6), that drive the accumulation of neutrophils and the inhibition of cytotoxic function of T cells. The treatment with ICI inhibitors in this model was not effective, while the inhibition of IL6 via blocking antibody or the depletion of neutrophil leaded to increased T cell function and tumor growth control in mouse model of lung cancer (126). Finally, LKB1 deficient tumors, fail to activate 5’ AMP-activated protein kinase (AMPK), thus keeping mTORC1 active and abrogating energetic checkpoint control (128). Indeed, KL mutant tumors are characterized by a pro-growth metabolism, with high glucose and glutamine uptake and usage that could represent an important mechanism through which the anti-tumor immunity is altered (69, 72).

### 3.3 Alterations of the Cyclic GMP-AMP synthase – stimulator of interferon genes pathway in cancer cells

Constitutive genomic instability and genotoxic stress imposed by oncogene, DNA replication and metabolic alterations in the cancer cells leads to dsDNA accumulation in the cytoplasm. This cytoplasmic dsDNA accumulation can further be enhanced by chemotherapy and radiotherapy induced genotoxic stress. The accumulation of cytoplasmic dsDNA determines the activation of the cGAS-STING pathway. The cGAS-STING pathway was initially described as an antiviral and anti-intracellular bacteria innate immune signaling pathway driving the expression of Interferon regulatory factor 3 (IRF3)/nuclear factor kappa-light-chain-enhancer of activated B cells (NF-κB) transcription programs that are characterized by the expression of type I interferon (IFN), interferon stimulated genes (ISG) and other immune-stimulatory cytokines. Thus, self and non-self-sources of cytosolic dsDNA can trigger cGAS/STING signaling in a very large variety of cell-types including immune cells, endothelial cells, fibroblasts, epithelial and cancer cells (129, 130).

cGAS catalyzes the formation of cyclic-di-guanosine-adenosine monophosphate (cGAMP) by degrading cytosolic dsDNA, then, cGAMP binds to and activates STING dimers which translocate from the endoplasmic reticulum toward their degradation into lysosomes while activating the Tank-Binding Kinase-I (TBK1) phosphorylation cascade (131). Ultimately, STING signaling drives IRF3 phosphorylation, dimerization, and translocation into the nucleus through TBK1 (132). Although this remains less characterized, STING mediated TBK1 and/or IKKe activation could lead to both, canonical (133) and non-canonical (134) NFκB signaling in immune and non-immune cells.

The activation of the cGAS/STING pathway in cancer cells seems to have controversial effects. Indeed, despite having high amount of dsDNA in their cytoplasm, type I IFN is hardly produced in cancer cells at the basal level without other stimulations (135), suggesting that cGAS-STING pathway may be somehow perverted in these cells as a mechanism of escape from immune sensing. As illustrated by mutant HRas^V12^ expression in human lung fibroblasts (IMR90) and mouse embryonic fibroblasts, oncogenic stress leads to dsDNA accumulation in the cytoplasm. This drives the expression of a senescence-associated secretory program (SASP) characterized by IL6, tumor necrosis factor (TNF), Chemokine (C-C motif) ligand 5 (CCL5)/RANTES and neutrophil attracting ELR-chemokines expression together with cell proliferation inhibition in a cGAS and STING dependent manner (129, 136) [for review see (137)]. cGAS/STING dependent senescence induction requires p38-Mitogen-activated protein kinases1 (MAPK) and p21 function and was posed as an important mechanism involved in chromosome stability (138). Besides cell senescence and chronic inflammation, cGAS/STING signaling in the cancer cell was also proposed to induce anti-tumor immunity. Indeed, STING expression is repressed in a variety of cancers (139–141). Particularly, in KL mice model, loss of LKB1 leads to cytoplasmic dsDNA accumulation released from aged/damaged mitochondria. Thus, KL tumors displaying low STING expression avoid constitutive ISG induction. Interestingly, an increased serine utilization and synthesis of S-adenylmethionine (SAM) in KL tumors that is associated with STING loss, and hyper activation of DNA (cytosine-5)-methyltransferase 1 (DNMT1) and enhancer of zeste homolog 2 (EZH2) epigenetic modifiers that use SAM as substrate could be responsible for STING promoter hyper-methylation necessary for lung tumor development and growth (141). STING activity could also be inhibited trough different mechanisms such as cytoplasmic dsDNA degradation by Trex1 (142), or post transcription modification limiting STING and TBK1 interaction (143) or by facilitating its degradation (144). However, the importance of each of these different levels of STING signaling regulation remains to be explored in the context of advanced cancer and ICB treatment.

More importantly, even in absence of functional STING, cGAS-dependent production of cGAMP in the different cancer cell types was proven to be crucial for the activation of an efficient antitumor immune-response both *in vitro* and *in-vivo* (135, 145). Indeed, cGAMP would be transferred *via* gap junctions to DCs, macrophages and non-tumor host cells, where STING activation will promote the production of immunostimulatory type I IFN (135) and therefore a good response to immunotherapy (146). The importance of cGAS was further illustrated by the fact that its expression correlates with immune activation and patients’ survival in melanoma (145). To avoid the immune response associated to cGAMP production and release, cancer cells can downregulate cGAS expression, as suggested in colorectal adenocarcinoma (139) and melanoma (140). Another possibility for cancer cell to escape STING-dependent immune surveillance was shown in breast cancer model. In this model, the ectonucleotidase ENPP1 is responsible for the degradation of extracellular cGAMP into extracellular AMP that can then be converted into immunosuppressive adenosine via CD73 (147),. Coherently, expression of ENPP1 in human cancer is associated with poor immune infiltration and resistance to PD1/PD-L1 immunotherapy (147). Thus, the cGAS/STING pathway represent a very attractive opportunity to increase anti-tumor immunity and cancer cell adjuvanticity. However, together with cell transformation, cancer cells acquire the ability to circumvent and/or subvert to their advantage the function of STING. In fact STING expression and activity in cancer was also associated with pro-tumor functions. A non-canonical STING pathway have been suggested to foster tumor progression (148), metastasis formation (134, 149), resistance to therapies (150), sustain the stemness traits of cancer cells (151) and escape immune control (148, 152). These evidences point out to a certain plasticity of STING pathway, that has not totally been uncovered and that cancer cells could take advantage of to escape from the immune system and at the same time fuel their growth.

To date, STING agonists have been developed to be injected intra-tumoral and fuel immune response directly *in situ*. Such administration led to tumor regression in mice model of melanoma, breast and colorectal cancer (153) and are expected to enhance ICB efficacy. However, intra-tumoral injection is not always possible, therefore other strategies are in development and several clinical assays are actually ongoing to test the efficacy of STING agonists alone or as adjuvant for ICB [recently reviewed by Amouzegar and colleagues (154)].

## 4 Discussion

All along this review, we have reported important characteristics linked with cancer cell immunogenicity and adjuvanticity. Then, we presented some mechanisms through which the cancer cell can adapted and escape from the pressure of the immune system. In this review article, we only focused on pathways and mechanisms occurring naturally in the cancer cells rather than those induced upon treatment. Hence, we omitted notions of immunogenic cell death that can be induced by chemotherapy and radiotherapy (155, 156). Altogether, discoveries summarized in this manuscript are pointing to the possibility that under ICB, cancer cell displaying specific alterations might be selected giving rise to the emergence of highly immune resistant cancer cell clones. However, during disease initiation and progression, tumor development occurs in the presence of a competent immune system in most of cancer patients, thus, it is also possible that ICB resistance mechanisms naturally pre-exist and that ICB only marginally influences cancer cell clones diversity.

We have seen that TAA and TSA load in the cancer cells dictate the quality of the ICB response, but for being effective as adaptive anti-tumor immunity targets, these tumor antigens have to be presented on the MHC-I molecules expressed by the cancer cells. An important study performed on melanoma by Zaretsky et al. in 2016 compared genetic alterations over four patients before and after disease relapse upon anti-PD-1 blockade (157). This allowed for the first time the identification of mutations associated with acquired resistance to ICB and shown loss of function mutation in IFN signaling (Janus kinase-1 and 2) in two patients and beta-2-microglobulin (B2M) in a third patient in ICB resistant cancer lesions (157). IFN signaling exert an antiproliferative effect on the cancer cells but also contributes to MHC-I induction while B2M expression is required for MHC-I expression and antigen presentation. Thus this study suggested in 3 patients over four that developed acquired resistance to ICB, MHC-I expression and then tumor antigen presentation by the cancer cell was impaired (157). Again, in line with the concept that acquired resistance to ICB relies and cancer cell loss of immunogenicity, Anagostou et al. showed the tumor antigens landscape evolves with acquired resistance to ICB in NSCLC (158). They observed that over 4 NSCLC patients, acquired resistance to ICB was linked with the loss of putative mutation associated with TSA expression through selection of cancer cell clones that do not presented these genetic alterations during the treatment course. These TSA were able to induce autologous T cell clonal expansion and the TCR clonality changed in these patients while ICB resistant cancer cell clones emerged (158). Thus, similarly to the cancer cell editing process occurring during disease appearance (159), ICB treatment might lead to the selection of cancer cell clones harboring lower immunogenic TSA load and presentation capability.

Cancer cells diversity can be assessed by single-cell RNA sequencing. In small cell lung cancer (SCLC) this approach allowed the identification of a stem-like, pro-metastatic tumor cell sub-population enriched at different prevalence across all patients analyzed and associated with worst survival. This population of cancer cells was characterized by the expression of PLCG2 and associated with a highly immunosuppressive TME (160). There are multiple studies in lung cancer and other pathologies showing that partial epithelial-to-mesenchymal transition phenotype of the cancer cell could be considered as a hallmark of cancer cell plasticity and refractoriness to ICB in patients (161, 162) and in mice (163). EMT signature in cancer cells links with the WNT/β-Cat pathway activation (164), cancer stem cell phenotype and multidrug resistance (165). Thus, it becomes critical to determine if ICB treatment might favor the selection of cancer cell sub-populations demonstrating strong EMT and stemness properties that would contributes to disease relapse and progression.

To conclude, while multiple important characteristics of the cancer cells were identified as essential for sustained ICB efficacy as well as refractoriness, some recent evidences indicate that upon ICB the cancer cell clones diversity is rearranged through the selection of transformed cells displaying lower immunogenicity. However, yet it is impossible to conclude whether ICB could also significantly change the behavior of the cancer cells toward the acquisition of a more aggressive phenotype such as EMT or to drive the emergence of cancer stem cell populations that would then be responsible for disease recurrence. Further research is required to determine how ICB efficacy might be influenced, not only by cancer cell clones diversity but also by the plasticity of the transformed cells present in the primary tumor and at metastatic sites. Such investigation will most probably help predicting patients’ response and proposing novel combination therapies to further increase the benefit of ICB in clinic.

## Author contributions

CU identified relevant literature and wrote the manuscript. CM contributed in identifying relevant literature and edited the manuscript. LG and NB edited the manuscript and identified relevant publications. JF supervised manuscript preparation, identified relevant literature, wrote and edited the text. All authors contributed to the article and approved the submitted version.

## Glossary

**Table:**

ACC	Acetyl CoA Carboxylase
aeTSA	Aberrantly expressed tumor specific antigen
AMP	Adenosine monophosphate
AMPK	AMP-activated protein kinase
ATF3	Activating transcription factor 3
ATF4	Activating transcription factor 4
ATP	Adenosine triphosphate
CAFs	Cancer associated fibroblasts
cGAMP	Cyclic di-guanosine-adenosine monophosphate
cGAS	Cyclic GMP-AMP synthase
COX-2	Cyclo-oxygenase-2
CRC	Colorectal cancer
CTLA-4	Cytotoxic T-lymphocyte associated protein 4
DC	Dendritic cell
DDR	DNA damage repair
dMMR	Deficient mismatch repair
DNA-PK	DNA-dependent protein kinase
DNMT1	DNA methyltransferase 1
dsDNA	double-stranded DNA
dsRNA	double stranded RNA
EGFR	Epidermal Growth Factor Receptor
ELR	Glutamate-Leucine-Arginine motif
ENPP1	Ectonucleotide pyrophosphatase/phosphodiesterase 1
ERK	Extracellular-signal related kinase
ERVs	Endogenous retroviruses
EZH2	Enhancer of zeste 2 polycombe repressive complex 2 subunit
FASN	Fatty acids synthase
Foxp3	Forkead box P3
G-CSF	Granulocyte-Colony Stimulating Factor
GLUT1	Glucose transporter 1
GM-CSF	Granulocyte-monocyte colony stimulating factor
HER-2	Human epithelial growth factor receptor-2
HIF-1α	Hypoxia-inducible factor-1α
MHC-I	Major histocompatibility complex-1
HK2	Hexokinase 2
HNRNPC	Heterogeneous nuclear ribonucleoprotein C
HNSCC	Head and neck squamous cell carcinoma
HO-1	Heme oxygenase-1
HRR-BER	Homologous recombination repair and base excision repair
HRR-MMR	Homologous recombination repair and mismatch repair
ICB	Immune checkpoint blockers
IDO-1	Indoleamine 2
3-dioxygenase-1	
IFN	Interferon
IKKe	Inhibitor of NFkB kinase subunit epsilon
IL-6	Interleukin-6
IL-17	Interleukin-17
IRF1	Interferon regulatory factor 1
IRF3	Interferon regulatory factor 3
ISG	Interferon stimulated genes
JNK	Jun N-terminal kinase
KL	KRAS-LKB1 co-mutated
KP	KRAS-TP53 co-mutated
LDH-A	Lactate dehydrogenase-A
LINEs	Long interspersed nuclear elements
lncRNA	Long non-coding RNA
lncRNA-AS	Long non-coding RNA antisense
MAGE-A3	Melanoma-associated antigen family member 3
MAPK	Mitogen-Activated Protein Kinase
MDA5	Melanoma differentiation-associated gene 5
MDSCs	Myeloid-derived suppressor cells
MSH2	mutS homolog 2
MUC-1	Mucin-1
MSl^hi^-CRC	Microsatellite-instability-high colorectal cancer
MSS-CRC	Microsatellite stable colorectal cancer
mTOR	mechanistic Target Of Rapamycin kinase
mTORC1	mTOR complex 1
NFkB	Nuclear factor kappa B
NK	Natural Killer
NRF2	Nuclear factor erythroid 2 related factor 2
NSAID	Non-steroidal anti-inflammatory drugs
NSCLC	Non-small cell lung cancer
NY-ESO-1	New York Esophageal cell carcinoma 1
ox-LDL	oxidized low-density-lipoproteins
PAP	Prostatic acid phosphatase
PD-1	Programmed cell death-1
PD-L1	Programmed cell death-ligand 1
PGE2	Prostaglandine-E2
PI3K	Phosphatidylinositol-3-kinase
PMN	Polymorphonuclear cells
POLD1	DNA polymerase delta 1
POLE	DNA polymerase epsilon
PTEN	Phosphatase and tensin homolog
RBPs	RNA binding proteins
RCC	Renal cell carcinoma
RIG-I	Retinoic acid-inducible gene I
SAM	S-adenosylmethionine
SASP	Senescence-associated secretory program
SCLC	Small cell lung cancer
SHP2	Src homology-2 domain containing phosphatase 2
SINEs	Short interspersed nuclear elements
STING	Stimulator of interferon genes
STK11	Serine/Threonine Kinase 11
TAAs	Tumor associated antigens
TBK1	Tank-Binding Kinase 1
TCA	Tricarboxylic acid
TCGA	The Cancer Genome Atlas
TCR	T-cell receptor
TEs	Transposable elements
TERT	Telomerase reverse transcriptase
TGF-β	Transforming Growth Factor-β
Th1	T-helper-I
TKI	Tyrosine kinase inhibitors
TLR3	Toll-like receptor 3
TLS	Tertiary-lymphoid structures
TMB	Tumor mutation burden
TME	Tumor microenvironment
TNB	Tumor neoantigen burden
TNF	Tumor Necrosis Factor
TNM	Tumor Node Metastasis
Treg	Regulatory T lymphocytes
TSA	Tumor-specific antigens
VEGF	Vascular Endothelial Growth Factor
WT	Wild type
